# Non-needle acupoint stimulation for prevention of nausea and vomiting after breast surgery: A meta-analysis

**DOI:** 10.1097/MD.0000000000014713

**Published:** 2019-03-08

**Authors:** Ran Sun, Wei Dai, Yang Liu, Changli Liu, Yongning Liu, Ying Gong, Xiaohong Sun, Tieying Shi, Mingzhi Song

**Affiliations:** a Department of Nursing; b Operation room, the First Affiliated Hospital of Dalian Medical University; c Health management and physical examination Center, the Affiliated Hospital of Qingdao University, Qingdao, Shandong; d Department of Orthopaedics, the First Affiliated Hospital of Dalian Medical University, Dalian, Liaoning; e Department of Orthopaedics, the Third Affiliated Hospital of Dalian Medical University, Jinpu New Area, Liaoning, People's Republic of China.

**Keywords:** acupoint, breast, meta-analysis, nausea, non-needling stimulation, PONV, vomiting

## Abstract

**Background:**

Breast disease has been a global serious health problem, among women. Surgery is the main treatment for the patients suffering from breast disease. Postoperative nausea and vomiting are still disturbing. Acupoint stimulation, an effective treatment of traditional Chinese medicine, has been used to reduce postoperative nausea and vomiting. Recently, non-needle acupoint stimulation becomes a new intervention. Though several clinical trials have been done, there is still no final conclusion on the efficacy. This Meta-Analysis aims at evaluating the efficacy of non-needle acupoint stimulation for prevention of nausea and vomiting after breast surgery.

**Methods:**

Systematic searches were conducted in PubMed, Embase, Cochrane, and Wanfang Med Online databases for studies. The review period covered from the inception of databases to December 31, 2017. The outcome measures of interest were frequency of nausea, frequency of vomiting, frequency of PONV, verbal rating scale of nausea, and use of rescue antiemetic. Data extraction and risks of bias evaluation were accomplished by 2 independent reviewers using the Cochrane Collaboration Review Manager software (RevMan 5.3.5).

**Results:**

Fourteen randomized controlled trials with a total of 1009 female participants in the non-needle acupoint stimulation group and control group met the inclusion criteria. Although the therapeutically effect on vomiting within postoperative 2 hours was not obvious, non-needle acupoint stimulation still had an important role in reducing nausea and vomiting within postoperative 48 hours. According to Jadad scale, there was moderate quality evidence for the pooled analysis results in this study. In addition, stimulating acupoint by wristband acupressure was more likely to cause adverse reactions.

**Conclusion:**

Non-needle acupoint stimulation can be used for female patients undergoing breast surgery to reduce postoperative nausea and vomiting. Into consideration, we recommend transcutaneous acupoint electrical stimulation on PC6 from 30 minutes before induction of anesthesia to the end of surgery for application. This non-pharmaceutical approach may be promising to promote the recovery of patients after breast surgery.

## 1 Introduction

Due to advances in diagnostic techniques, diagnosis of breast disease is getting easier.^[1]^ As the main part of this disease, breast cancer is one of the most common causes of death among women.^[2]^ From that, breast disease has been a global serious health problem. Surgery seems to be the only alternative for the patients suffering from most of breast cancers, some kinds of benign breast diseases, and breast reconstruction. Although the curative effect of breast surgery is improving deeply, postoperative complications are still disturbing.

Among the postoperative complications, nausea and vomiting are important ones. Currently, the term “postoperative nausea and vomiting (PONV)” is often defined as nausea or vomiting within 24 hours after surgery. It has an incidence of 20% to 30% after anesthesia.^[3]^ Current thinking suggests that four primary risk predictors including female gender, history of motion sickness, nonsmoking, and the use of postoperative opioids are related to the PONV.^[4]^ Method and duration of anesthesia, and medications used during the surgical procedures are also known be related to PONV.^[1,5]^ Due to the gender specificity, breast surgery is often followed by a high incidence of PONV. Some reports showed that about 47% to 80% of patients undergoing minor breast surgery, mastectomy, and breast reconstruction suffer from PONV.^[5–7]^ Except for discomfort feelings, PONV can lead to aspiration, wound dehiscence, hematoma, bleeding, dehydration, exhaustion, electrolyte imbalance, mobilization delay, slow recovery, and disability to begin oral medications.^[8]^ In addition, PONV increases health care costs including prolonging hospital stay and unexpected hospitalization of outpatient surgery patients.^[7,9,10]^ Although application of antiemetic drugs is widespread, the side-effects including headache, constipation, and abdominal pain always promotes the patients’ suffering.^[11]^ Non-pharmaceutical and safe method is better suited to the requirements of current medical development. Therefore, the new settlement of PONV becomes critical for patients undergoing breast surgery. Unfortunately, the best answer is still in the pending.

Traditional Chinese medicine (TCM), especially acupuncture, plays a positive role in prophylaxis and treatment of PONV.^[12,13]^ It has been confirmed that acupoints such as Neiguan (PC6), Laogong (PC8), Waiguan (SJ5) Zusanli (ST36), Hegu (LI4), and Quchi (LI11) can be used for reducing PONV.^[14–27]^ The basic theory of action mechanism is related to activating meridians by acupuncture.^[28]^ Acupoints could be stimulated by acupuncture needle, electrical stimulation, and acupressure and so on. The use of traditional acupuncture was subject to the treatment experience. However, with the continuous simplification and commercialization, non-needle acupoint stimulation including electroacupuncture and acupressure are gradually substituted for the traditional acupuncture and have been common nursing methods. Effectively reducing the incidence of postoperative complications, electroacupuncture, and acupressure instruments have been widely applied to patients in the clinical nursing work. Major therapeutic purposes of non-needle acupoint stimulation include reducing PONV, decreasing postoperative pain and improving gastrointestinal function.^[29–31]^ The effect of acupoint stimulation on reducing PONV is studied most, mainly involving breast surgery, abdominal surgery, caesarean section and thyroidectomy.^[13,15,31,32]^ Since the new method was introduced to the recovery after breast surgery, several clinical trials have been done for unveiling the truth. However, there is still no final conclusion on the efficacy of non-needle acupoint stimulation as an intervention for reducing PONV. Here, we performed this review of the included studies to reveal the role of non-needle acupoint stimulation on PONV.

## 2 Methods

This review was reported according to the PRISMA statement.^[33]^ Ethical approval was not necessary for this review study.

### 2.1 Database and search strategy

**Figure 1:**
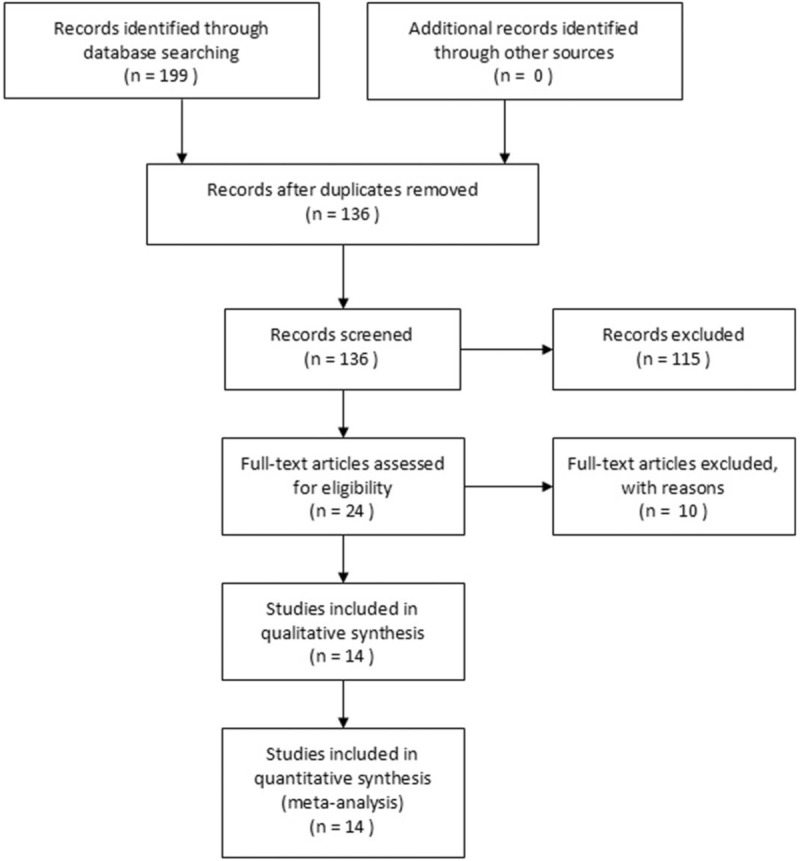
Flow diagram of the study selection process.

We searched the following databases from their inception through December 31, 2017: PubMed, Embase, Cochrane and Wanfang Med Online databases. The following search terms were used individually or jointly: “acupoint”, “acupuncture point”, “breast”, “nausea”, “vomiting”, “postoperative complication”and “recovery”. No language restrictions were imposed. In addition, experts in this field were consulted to search for all relevant studies, and the reference lists of all these included studies and reviews were manually searched for other articles. The search strategy for each database is available in Figure 1.

### 2.2 Inclusion criteria

The studies which involved electrical stimulation or acupressure as a therapeutic intervention for preventing postoperative nausea and vomiting after breast surgery were included. All included studies should meet the following criteria:

(1) studies that had a randomized controlled trial (RCT) design and data collected after the surgery;
(2) electrical stimulation or acupressure was the primary intervention in the treatment group;
(3) sham and/or active control procedure was performed;
(4) primary outcomes included frequency of nausea, frequency of vomiting, frequency of PONV, verbal rating scale (VRS) of nausea, use of rescue antiemetic.

Exclusion criteria were as below:

(1) RCTs including other patients besides who underwent breast surgery;
(2) RCTs using antiemetic, laser acupuncture, traditional acupuncture and massage as controls;
(3) RCTs using traditional acupuncture as the primary interventions in the treatment group;
(4) trials which failed to offer proper data to extract.

### 2.3 Data extraction

Two reviewers (Wei Dai, Changli Liu) independently evaluated and extracted data from all included studies using a standardized extraction form especially created for this meta-analysis (Table 1). The form contained information of author, country, participants, the methodological aspects of the study, interventions, and measured outcomes. Additionally, the methodological quality of the studies included in this meta-analysis was evaluated using the Jadad scale.^[34]^ Perfect randomization, proper blinding, and adequate descriptions of withdrawals and dropouts were considered to assess the included studies. These 2 individual forms were discussed by all the reviewers until a consensus was reached, and these forms were merged into a single extraction form. Persistent disagreements were settled by. When necessary, the authors of these included studies were contacted for further information. Disagreements were resolved by a third reviewer (Mingzhi Song). When necessary, missing data or further information was sought from the primary authors via email if necessary.

**Table 1:**
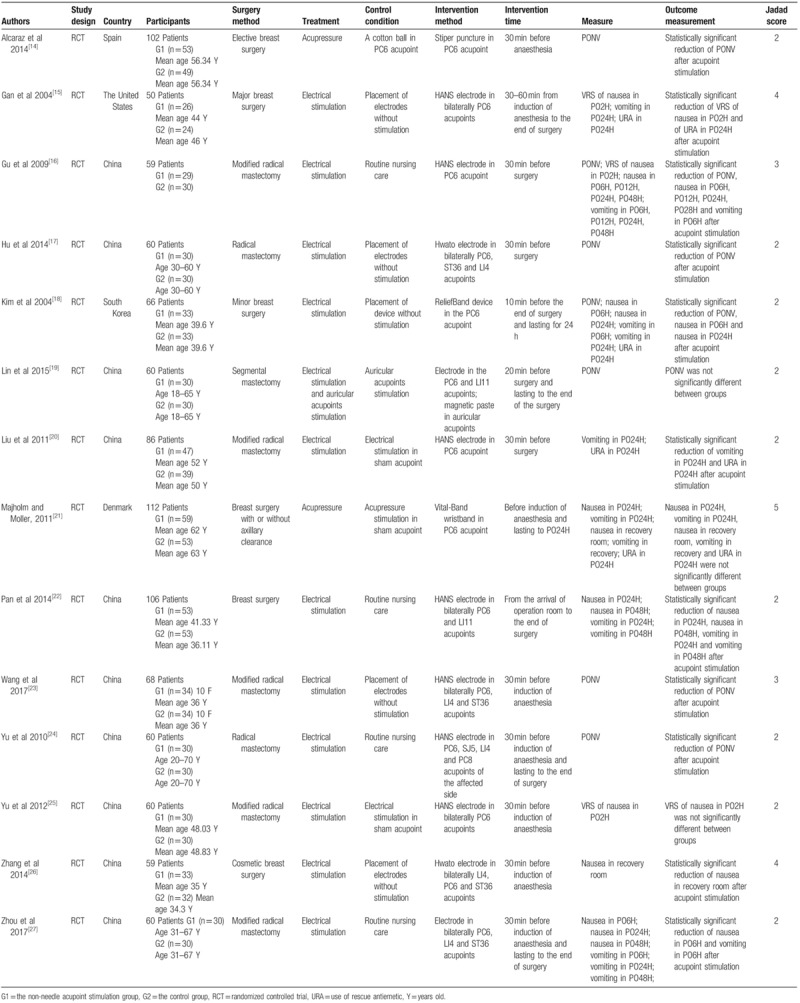
Characteristics of the included studies.

#### 2.3.1 Assessment for risk of bias

Risk of bias assessment of the included RCTs was independently fulfilled by 2 reviewers (Wei Dai, Changli Liu) using the Cochrane criteria.^[35]^ Discrepancies were resolved by discussion. Risk of bias of the following domains were assessed random sequence generation (selection bias), allocation concealment (selection bias), binding of participants and personnel (performance bias), binding of outcome assessment (detection bias), incomplete outcome data (attrition bias), selective reporting (reporting bias) and other potential sources of bias. Here, due to the characteristic of acupoint stimulation RCTs, other bias was defined as whether the study introduced the detailed operation method. Three levels were used to evaluate the trials: low risk of bias (all the items were in low risk of bias), high risk of bias (at least 1 item was in high risk of bias) and unclear risk of bias (at least 1 item was in unclear risk of bias). For examples, as for “low risk” of bias for the domain of random sequence generation, the investigators should describe a random component in the sequence generation process such as referring to a random number table; using a computer random number generator or coin tossing.

### 2.4 Statistical analysis

Meta-analysis was performed by using Review Manager V5.3. Continuous data were presented as mean difference (MD) and its 95% confidence intervals (CI). Heterogeneity was examined using the *I*^2^ test, where *I*^2^ values of 50% or more were considered to be indicative of a substantial level of heterogeneity.^[35]^ Random effects model was employed to present MD. Based on different outcome measures, if significant heterogeneity between studies was detected, the subgroup analysis would be conducted to investigate possible causes from clinical perspectives. Regarding dichotomous data, results were presented as RRs with 95% CIs, using random-effects model.

## 3 Results

### 3.1 Literature search

The process of literature search was shown in Figure 1. A total of 199 potential trials were identified via the primary search strategy. After duplicates remove, 63 studies remained. Twenty-four potentially relevant studies were evaluated for eligibility. Then, 10 studies with unavailable data were excluded. Finally, Fourteen RCTs were included in the current meta-analysis.^[14–27]^

### 3.2 Study characteristics

The characteristics of the 14 studies that met the inclusion criteria were shown in Table 1. This analysis included a total of 1009 female patients. And the sample size ranged from 50 to 112. And 10 included studies were conducted in China,^[16,17,19,20,22–27]^ 1 in Spain,^[14]^ 1 in the United States,^[15]^ 1 in South Korea^[18]^ and 1 in Denmark.^[21]^

Two patterns of non-needle acupoint stimulation were included in these studies: electrical stimulation (N = 12 trials) and acupressure (N = 2 trials). The most frequently used acupuncture points were PC6 (N = 14 trials), LI4 (N = 5 trials), ST36 (N = 4 trials), LI11 (N = 2 trials) and SJ5 (N = 1 trial). Moreover, seven trials adopted bilateral acupoints stimulation and others performed the unilateral acupoints stimulation. Participants received acupoints stimulation more than 30 minutes. All the trials began the intervention before surgery and most of the intervention stopped at the end of surgery.

Methods of control groups consisted placement of sham stimulation (N = 6 trials), stimulation in sham acupoint (N = 3 trials), routine nursing care (N = 4 trials) and auricular acupoints stimulation (N = 1 trial).

Nausea and vomiting were measured by frequency of PONV, frequency of nausea (PO6H, PO12H, PO24H, and PO48H), frequency of vomiting (PO6H, PO12H, PO24H, and PO48H), frequency of nausea in recovery room, frequency of vomiting in recovery room, VRS and use of rescue antiemetic.

Furthermore, in these studies, all the included participants underwent general anesthesia.

### 3.3 Risk of bias assessment

**Figure 2:**
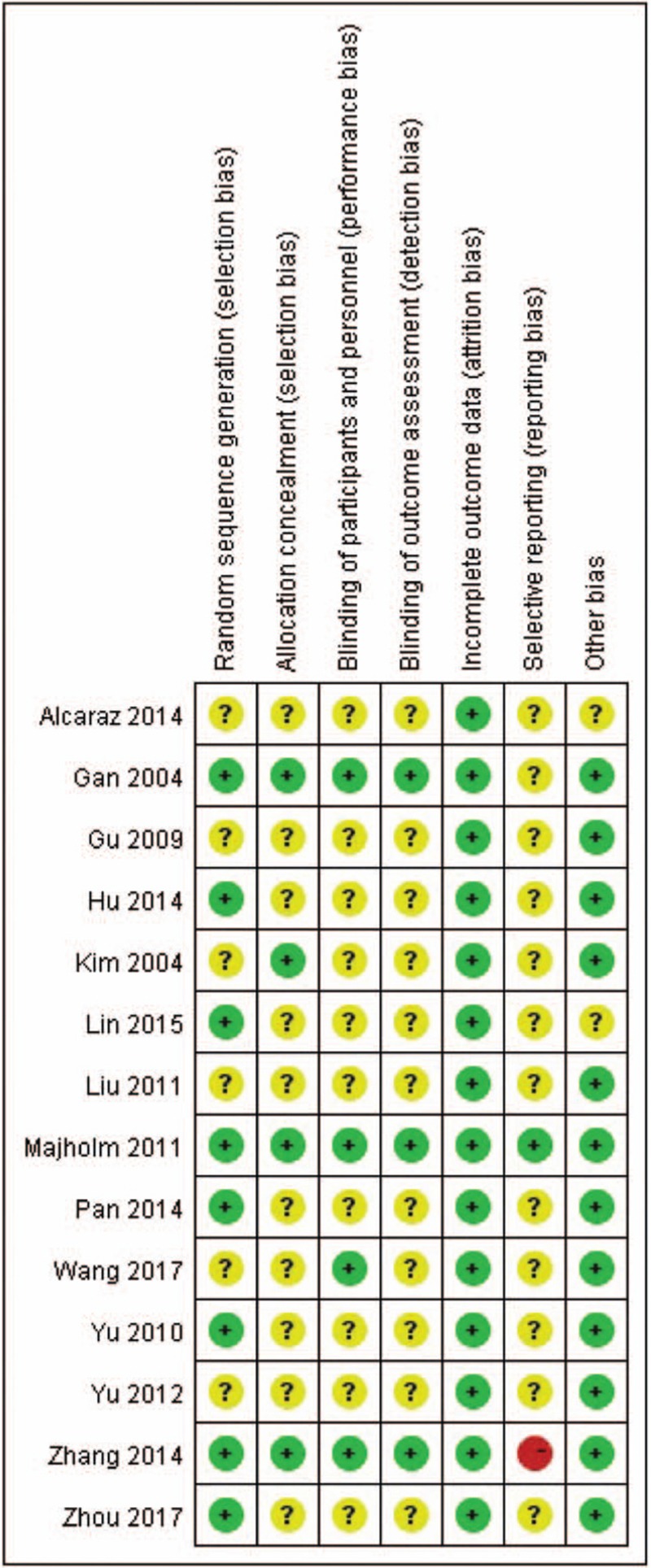
Risk of bias for included trials.

Figure 2 summarized the risk of bias in the included studies. Randomization was performed in all 14 RCTs. Four studies adopted random number table.^[17,24,26,27]^ Two studies used opaque envelopes.^[19,21]^ One study performed the random number generator.^[15]^ One used SPSS software to generate random numbers.^[22]^ The randomization methods of other trials could not be obtained from the articles. Three studies introduced allocation concealment.^[15,21,26]^ Due to the inaccessibility of the trail protocol, reporting bias was generally “unclear” in the included RCTs. Only 2 trail protocols can be retrieved with the register numbers.^[21,26]^ One trial^[21]^ was considered as having a low risk of reporting bias while another^[26]^ was elected as “high risk”. There were only 4 trials^[15,21,23,26]^ performed a double-blinded method, because of the characteristics of acupoint stimulation. Two trials^[16,21]^ reported 23 dropouts, and only 1 of them provided no details on its dropout. Two trials^[20,22,27]^ were considered as having an unclear risk of other bias because of the lack of detailed description on non-needle acupoint stimulation.

### 3.4 Meta-analysis outcome

**Figure 3:**
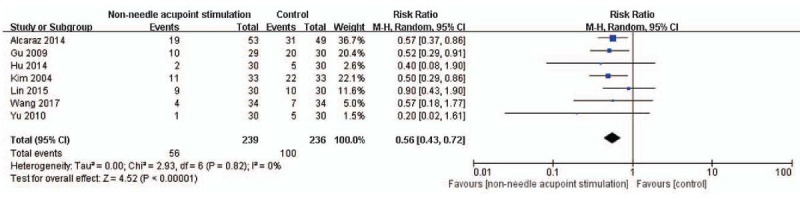
The forest plot indicates the difference of PONV between the non-needle acupoint stimulation group and control group. RevMan 5.3 software (Cochrane Information Management System) was used for this meta-analysis.

**Figure 4:**
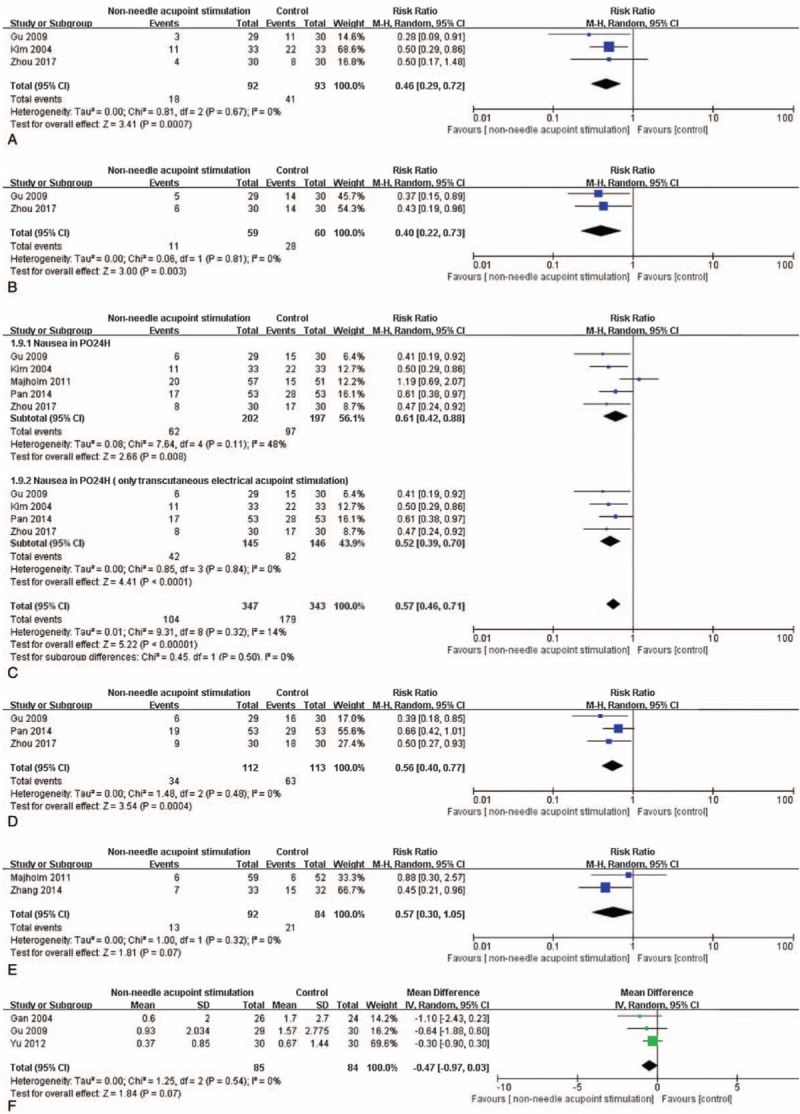
The forest plots indicate the differences in nausea within PO6H (A), nausea within PO12H (B), nausea within PO24H (C), nausea within PO48H (D), and nausea in the recovery room (E).

**Figure 5:**
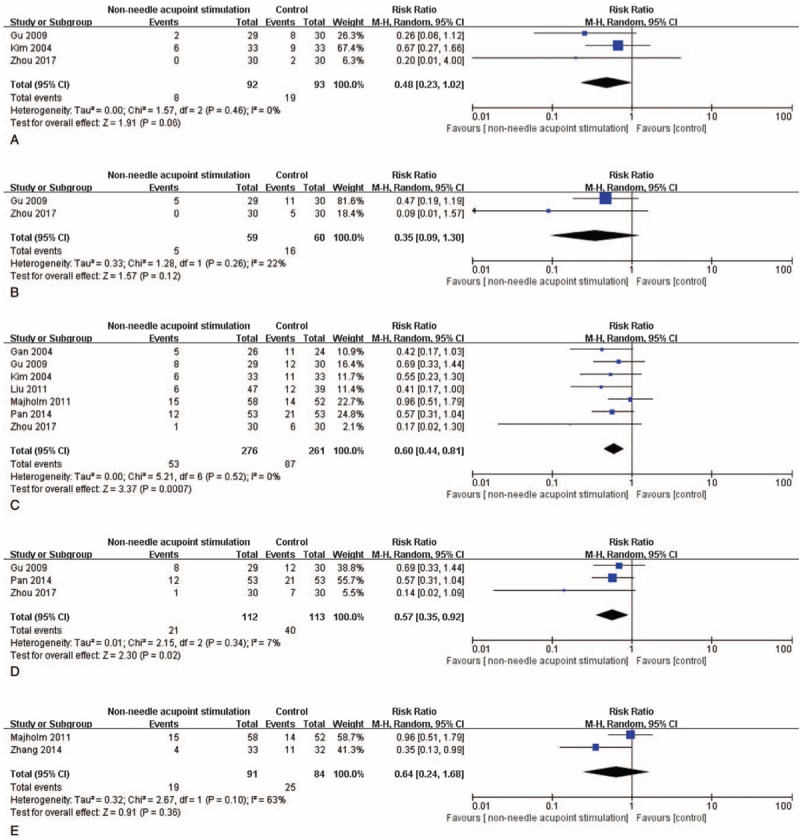
The forest plots indicate the differences in vomiting within PO6H (A), nausea within PO12H (B), vomiting within PO24H (C), vomiting within PO48H (D), VRS of nausea within PO2H (E), and nausea in the recovery room (F).

**Figure 6:**
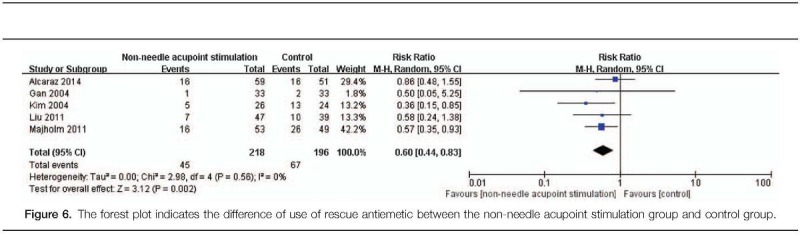
The forest plot indicates the difference of use of rescue antiemetic between the non-needle acupoint stimulation group and control group.

Based on various outcome measures after non-needle acupoint stimulation and control of the included studies, different pooled data of 14 RCTs were used in meta-analysis, respectively. The effect estimates of non-needle acupoint stimulation were shown in the forestplots (Figs. 3–6).

### 3.5 PONV

Half of the included studies measured and recorded the results of PONV after non-needle acupoint stimulation. Seven trials (N = 475)^[14,16–19,23,24]^ reported that non-needle acupoint stimulation might more effectively reduce PONV, compared with the control (RR: 0.56, 95% CI: 0.43–0.72, N = 475, and *I*^2^ = 0%; Fig. 3).

### 3.6 Nausea

#### 3.6.1 Nausea within PO6H

Nausea within PO6H in non-needle acupoint stimulation groups was compared with that in the control groups. Data from 3 studies^[16,18,27]^ indicated that nausea during this period (N = 185) had an evidence of significant difference (RR: 0.46, 95% CI: 0.29–0.72, N = 185, and *I*^2^ = 0%; Fig. 4A). This result indicated that non-needle acupoint stimulation could reduce nausea within PO6H compared with that in the control group.

#### 3.6.2 Nausea within PO12H

There was a significant difference about the results of nausea within PO12H. The combination of these 3 trials^[16,27]^ revealed that non-needle acupoint stimulation might more favorably reduce nausea within PO12H (RR: 0.40, 95% CI: 0.22–0.73, N = 119, and *I*^2^ = 0%; Fig. 4B).

#### 3.6.3 Nausea within PO24H

The analysis of data obtained from five studies^[16,18,21,22,27]^ indicated heterogeneity with respect to nausea within PO24H (*P* < .05, *I*^2^ = 49%). The decreasing change in non-needle acupoint stimulation group was statistically significant (RR: 0.61, 95% CI: 0.42–0.88, N = 399, and *I*^2^ = 49%; Fig. 4C). In this result, there were 2 kinds of non-needle acupoint stimulation, but only 1 trial performed acupressure. So, when the trial by Majholm^[21]^ was excluded, the heterogeneity decreased to 0% (RR: 0.52, 95% CI: 0.39–0.70, N = 291, and *I*^2^ = 0%, Fig. 4C). The remained trials revealed that, compared with control intervention, transcutaneous electrical acupoint stimulation could reduce nausea within PO24H compared with that in the control group.

#### 3.6.4 Nausea within PO48H

Three of the included studies^[16,22,27]^ measured nausea within PO48H of the participants (N = 226) after non-needle acupoint stimulation. There was a significant difference in nausea within PO48H (RR: 0.56, 95% CI: 0.40–0.77, N = 225, and *I*^*2*^ = 0%; Fig. 4D). This result indicated that nausea within PO48H could be improved by non-needle acupoint stimulation.

#### 3.6.5 Nausea in the recovery room

Two studies^[21,26]^ were pooled and analyzed for the results of nausea in the recovery room. There was not a significant difference of nausea between non-needle acupoint stimulation and the control intervention in the recovery room (RR: 0.57, 95% CI: 0.30–1.05, N = 176, *I*^2^ = 0%, *P* > .05; Fig. 4E).

#### 3.6.6 VRS of nausea within PO2H

Three studies^[15,16,25]^ measured nausea by using VRS in PO2H. With no statistical significance, this result revealed that non-needle acupoint stimulation could not relieve nausea within PO2H (MD:−0.47, 95% CI: −0.97–0.03, N = 169, and *I*^2^ = 0%, *P* > .05; Fig. 4F).

### 3.7 Vomiting

#### 3.7.1 Vomiting within PO6H

There were 3 studies^[16,18,27]^ recorded vomiting within PO6H after non-needle acupoint stimulation. Data obtained from these studies revealed that vomiting in PO6H (N = 186) had no statistical significance (*P* > .05). This result indicated that non-needle acupoint stimulation could not reduce vomiting within PO6H compared with that in the control group (RR: 0.48, 95% CI: 0.23–1.02, N = 185, and *I*^2^ = 0%; Fig. 5A).

#### 3.7.2 Vomiting within PO12H

The analysis of data obtained from 2 studies^[16,27]^ indicated low significant heterogeneity and no statistical significance with respect to vomiting within PO12H (*P* > .05, *I*^2^ = 22%). There was no evidence of significant difference in vomiting within PO12H (RR: 0.35, 95% CI: 0.10–1.2, N = 119; Fig. 5B).

#### 3.7.3 Vomiting within PO24H

There were 7 trials^[15,16,18,20–22,27]^ focus on results of vomiting within PO24H after non-needle acupoint stimulation. The combination of these data had an evidence of significant difference and revealed that non-needle acupoint stimulation might more favorably reduce vomiting within PO24H (RR: 0.60, 95% CI: 0.44–0.81, N = 537, and *I*^2^ = 0%; Fig. 5C).

#### 3.7.4 Vomiting within PO48H

Three studies^[16,22,27]^ were pooled and analyzed for the results of vomiting within PO48H. Data from these studies had a negligible statistically heterogeneity (*P* < .05, I2 = 3%). There was a significant difference of vomiting between non-needle acupoint stimulation and the control intervention during postoperative 48 hours (RR: 0.57, 95% CI: 0.35–0.92, N = 225; Fig. 5D). This result indicates that non-needle acupoint stimulation might improve vomiting within PO48H.

#### 3.7.5 Vomiting in the recovery room

Two of the included studies^[21,26]^ recorded vomiting of the participants (N = 175) in the recovery room. The results of non-needle acupoint stimulation groups were compared with that of the control groups (Fig. 5E). There was no evidence of significant difference in vomiting in PO12H (RR: 0.35, 95% CI: 0.10–1.2, N = 120, *I*^2^ = 0%, *P* > .05; Fig. 5E).

#### 3.7.6 Use of rescue antiemetic

The analysis of data obtained from 5 studies^[14,15,18,20,21]^ indicated a statistical significance with respect to use of rescue antiemetic within PO24H. This result showed that non-needle acupoint stimulation had a positive effect on reducing use of rescue antiemetic within PO24H compared with that in the control group (RR: 0.60, 95% CI: 0.44–0.83, N = 112, and *I*^2^ = 0%; Fig. 6).

### 3.8 Adverse events

Only 2 study reported adverse events in the intervention or control group. Majholm and Møller reported 77 patients with wrist and hand side effects such as redness, swelling, tenderness, and paresthesias that were caused by the wristbands.^[21]^

## 4 Discussion

Despite the fact that the curative effect of antiemetic on PONV has been widely proved, the avoidless side-effect remains a challenge to both surgeons and nurses.^[36]^ There are reports that the female patients undergoing different kinds of breast surgery have a stronger tendency to suffer from PONV.^[4]^ Facing these problems, various methods from clinical to nursing aspects such as surgery methods changing from mastectomy to modified radical mastectomy and from usual care to comfortable nursing have been applied to minimize the incidence of nausea and vomiting after breast surgery. However, the safe and effective method still remains unknown.

Acupoint stimulation is a complex, ritualistic somatosensory intervention with multiple components. Acupuncture is an original and important method of acupoint stimulation. Along with the development of TCM, more and more researchers apply acupoint stimulation to settle complications after treatment. Because of the particularity of acupuncture, reliance on skilled acupuncturist becomes the crucial part of treatment as well as limits the dissemination and generalization. The problem has been resolved, since instrumented replacements of acupuncture like transcutaneous electrical acupoint stimulation and acupressure are widely used to reduce postoperative complications. Particularly, studies about nausea and vomiting with satisfactory curative effects can often be found.^[37]^

According to TCM constitutional theory, most of patients undergoing breast surgery have Qi-stagnation constitution or Qi-deficiency constitution.^[38]^ And patients with PONV often have Qi-deficiency constitution. By stimulating acupoints, meridian and collaterals could be activated to tonify and promote Qi. Neiguan, for example, is an important acupoint of pericardium meridian, which has an effect on regulating Qi and decreasing inverse. Additionally, stimulating Laogong, Waiguan, Zusanli, Hegu or Quchi could also be used for reducing PONV by activating different meridians. Therefore, theoretically, acupoint stimulation may prevent nausea and vomiting after breast surgery. In response to this, some clinical researches have been done to explore practical effects.^[14–27]^ Unfortunately, conclusions from these researches are not the same. Comprehensive summary and analysis become more urgent.

Through rigorous reviewing and screening, we finally collected 14 trials to complete a meta-analysis to explore the result whether acupoint stimulation could have effect on reducing PONV after the breast surgery or not. In this review, unclear risk of bias was found in most of the included trails. Description of blind method and allocation concealment were the main reason that was prone to prevent the analysis of a subjective outcome. Jadad scale was applied to assess all selected trials, a high-quality study should have the Jadad score equal to or more than 3.^[34]^ All of included studies had the Jadad score no less than 2. There were nine trials with Jadad score 2. Combining particularity of acupoint stimulation and elaborative evaluation, reviewers found that design and implementation of these trials were reasonable and reliable. Therefore, these trials with Jadad score 2 were also included. These enrolling criteria allowed us a moderate quality analysis.

Methods of evaluating nausea and vomiting mainly include frequency, VRS of nausea, and use of rescue antiemetic. Here, the included outcomes were within PO48H. According to the results of analysis, nausea could be reduced by acupoint stimulation in the early phase after breast surgery (0-12H). However, acupoint stimulation had no reducing effect on vomiting at the same time. Nausea was often treated as the early symptom of vomiting. From the data of included studies, the frequency of vomiting in the early phase was less than other phases. This reasonably explained why pooled analysis did not support the effectiveness of acupoint stimulation for reducing vomiting. PO24H was thought to be the medium phase in this study. Frequency of PONV, frequency of nausea, and frequency of vomiting were selected to assess the curative effect of acupoint stimulation in the early and medium phases (0-24H). Analysis results kept consistency showing that acupoint stimulation was able to markedly reduce the occurrence of nausea and vomiting. In this period, frequency of nausea and vomiting increased. Although there was the difference of included patients, those research results about other kinds of surgery still supported our viewpoint.^[39]^ Analyzing the outcome of nausea within PO24H, we only found heterogeneity appearing in this result. Then, according to the classification of non-needle acupoint stimulation, a subgroup analysis was completed and showed no heterogeneity. Between electrical stimulation and acupressure, there may be some differences such as operation, curative effect and so on. This explained the reason of the existence of heterogeneity. Within PO48H, the possibility of nausea and vomiting continues to increase for postoperative patients. The pooled analysis of 2 outcomes including nausea and vomiting suggested the effect of non-needle acupoint stimulation on reducing nausea and vomiting after breast surgery continue until PO48H. In addition, adverse events were reported in only 1 included study, which adopted wristbands to stimulating acupoint. However, these commercialized wristbands caused redness, swelling, tenderness and paresthesias. The occurrence of adverse events was related to tight wearing.

In terms of the choice of non-needle acupoint stimulation, electrical stimulation was more popular. The most common acupoint is PC6 that was selected in all the included studies. This was in line with trends in research that often recommended PC6 as the acupoint to prevent nausea and vomiting. Acupoint selection could be either bilateral or unilateral. Meanwhile, other acupoints also could be chose to play a synergistic role of reducing nausea and vomiting. Working time was generally specified as from 30 minutes before induction of anaesthesia to the end of surgery. When electrical stimulation was selected, working electric current was different among various instruments. Without the need of professional acupuncturist, non-needle acupoint stimulation has been gradually popularized in clinical nursing practice and revealed the potential of replacing antiemetic. Moreover, there was an inevitable question that the use of acupressure is often limited to wristband structures that cause uncomfortable or tight feelings.^[40]^ The presentation of adverse events may lead to reduce the usage of acupoint stimulation by wristband. Therefore, transcutaneous electrical acupoint stimulation seems safer than acupressure by wristband acupressure.

Most of studies that were included in this review had unclear risk of bias, recruited a small number of patients, and provided sparse data on most of our pre-established outcomes of interest. So, the pooling results in these meta-analyses were affected. Heterogeneity existed in the aspects of type of surgery, duration of surgery and anesthesia. From the viewpoint of gastrointestinal stimulation and nervous stimulation, laparoscopic abdominal surgery, gynecological surgery, and procedures involving the ear, nose and throat were easier to induce PONV.^[41]^ However, the difference of increasing PONV among different breast surgeries could not be ignored. Duration of surgery always had effect on incidence of PONV.^[42]^ Here, due to the selection of different breast surgery, the duration of surgery varied greatly from study to study. Anesthetic agents were also related to PONV. The use of anesthetic inhalation agents like nitrous oxide has long been associated with an increase in the risk of PONV.^[41]^ On the contrary, total intravenous anesthetic using propofol has been found to reduce PONV.^[41]^ Therefore, heterogeneity in anesthetic agents was obvious in the included studies. Furthermore, there were only 2 studies about acupressure. Others selected electroacupuncture as the therapeutic method for PONV. For reducing PONV of breast surgery, the different between acupressure and electroacupuncture was no conclusion. But heterogeneity in acupoint stimulation methods could not be ignored, too.

To some extent, this had an effect on the final result of meta-analysis. The main methodological limitations of these included studies were the lack of description of allocation concealment, blinding of participants, and outcome assessors; which were due to the characteristic of non-needle acupoint stimulation. Due to the different publication standard, risk of bias in English trials was lower than that in Chinese trials. In addition, the inclusion criteria of patients in different studies was inconsistent, especially in age, history of nausea and vomiting, type of breast disease and surgery, the duration of surgery and use of anesthetic drug. Further studies with more consistent measurements and more standard data records would help to more accurately confirm the final conclusion.

## 5 Conclusion

The results of this meta-analysis of fourteen relatively studies showed that non-needle acupoint stimulation was an effective method for reducing nausea and vomiting of female patients after breast surgery. Because of the limited number and quality of included studies, this conclusion could not be considered a conclusive statement. As one of the most commonly used non-pharmaceutical therapy, acupoint stimulation was especially suitable for solving PONV by nurses. It could be recommended in patients undergoing breast surgery with moderate-high risk for PONV or drugs contraindications. The routine operation was application of transcutaneous acupoint electrical stimulation on PC6 from 30 minutes before induction of anesthesia to the end of surgery. The comparison among various methods and different stimulating acupoints will be a meaningful topic for future studies. Although the quantity of Chinese studies about acupoint stimulation mounted up, more well-designed studies should be carried out.

## Author contributions

Conceived and designed this meta-analysis: STY, SMZ and SR. Performed this meta-analysis: SR, DW and LCL. Analyzed the data: SMZ and SR. Contributed to software analysis and review of the paper: SR, DW, LCL, LYN, GY, SXH, STY and SMZ. Wrote the paper: SR and SMZ. Revised the paper: LY
